# Societal Age Stereotypes in the U.S. and U.K. from a Media Database of 1.1 Billion Words

**DOI:** 10.3390/ijerph18168822

**Published:** 2021-08-21

**Authors:** Reuben Ng

**Affiliations:** 1 Lee Kuan Yew School of Public Policy, National University of Singapore, Singapore 119260, Singapore; spprng@nus.edu.sg; 2 Lloyd’s Register Foundation Institute for the Public Understanding of Risk, National University of Singapore, Singapore 119260, Singapore

**Keywords:** ageism, age discrimination, aging narratives, media portrayals of aging, text as data, psychomics, quantitative social science

## Abstract

Recently, 194 World Health Organization member states called on the international organization to develop a global campaign to combat ageism, citing its alarming ubiquity, insidious threat to health, and prevalence in the media. Existing media studies of age stereotypes have mostly been single-sourced. This study harnesses a 1.1-billion-word media database comprising the British National Corpus and Corpus of Contemporary American English—with genres including spoken/television, fiction, magazines, newspapers—to provide a comprehensive view of ageism in the United Kingdom and United States. The US and UK were chosen as they are home to the largest media conglomerates with tremendous power to shape public opinion. The most commonly used synonym of older adults was identified, and its most frequently used descriptors were analyzed for valence. Such computational linguistics techniques represent a new advance in studying aging narratives. The key finding is consistent, though no less alarming: Negative descriptions of older adults outnumber positive ones by six times. Negative descriptions tend to be physical, while positive ones tend to be behavioral. Magazines contain the highest levels of ageism, followed by the spoken genre, newspapers, and fiction. Findings underscore the need to increase public awareness of ageism and lay the groundwork to design targeted societal campaigns to tackle ageism—one of our generation’s most pernicious threats.

## 1. Introduction

Proposed by Butler [1], the concept of ageism was originally conceived as comprising three distinct but interrelated components: prejudicial attitudes, discriminatory behavior, and institutional policies. Today, the term ‘ageism’ is more commonly understood as the stereotyping, prejudice and discrimination of people on the grounds of age [2]. In 2016, 194 World Health Organization (WHO) member states called on the international organization to develop a global campaign to combat ageism, citing its alarming ubiquity and insidious threat to health [3]. However, the ongoing COVID-19 crisis has brutally exposed the reality of ageism in society. Both older and younger people have been subject to various stereotypes in mainstream discourse; the constant homogenization of older adults as frail and vulnerable [4] has spawned distasteful references to the virus as a ‘Boomer Remover’ [5], while young people have been depicted as selfish and even blamed for spikes in COVID-19 cases [6].

Although ageism can be directed at people of all ages, the present study focuses on ageism towards older adults. Inaccurate and negative stereotypes of older adults have unfortunately resurfaced and intensified during the outbreak. Seeking to quantify the economic cost of ageism, Levy and colleagues [7] combined the impact of predictors of ageism—negative age stereotypes and self-perceptions of aging—with healthcare spending data for the eight most expensive health conditions ailing Americans aged 60 and above. They found that the one-year cost associated with ageism in the United States was $63 billion. It is therefore important to understand the types of age stereotypes that exist in society. Against this backdrop, the present study leverages a corpus of 1.1 billion words from multiple sources—newspapers, magazines, books, and television/radio transcripts—and applies computational linguistics to analyze the kinds of age stereotypes that prevail in the media.

Stereotypes refer to widely held beliefs about members belonging to a particular social group. Age stereotypes often include oversimplified generalizations about how people at, below or over a certain age ought to behave without any consideration of individual differences [8]. Stereotypes of old age can be positive, neutral, or negative. Positive age stereotypes refer to favorable beliefs about older adults (e.g., wise, kind, etc.), while negative age stereotypes refer to unfavorable beliefs about older people (e.g., slow, cranky, etc.). Past research has established that older adults who embrace positive stereotypes about aging are more likely to have better health outcomes than those who hold negative age stereotypes [9].

The role of the media in perpetuating age stereotypes has been widely acknowledged [10]. As a major agent of socialization, the media serves as a powerful avenue to understand how old age is socially constructed. Cultivation theory indicates that repeated exposure to the media can shape individuals’ perceptions of reality [11]. Portrayals of older adults in the media have the potential to reinforce stereotypes and shape individuals’ attitudes toward the aging process, ultimately predicting health outcomes in later life [12]. Media representations can be either visual or discursive in nature. The concept of ‘visual ageism’ refers to the practice of visually underrepresenting older adults or presenting them in a prejudiced manner [13]. Examples of visual ageism include the portrayal of older adults as lacking positive traits, as well as the non-realistic or exaggerated portrayal of this cohort [13]. Our study focuses on the discursive representations of older adults. It is well established in the social sciences that prejudice contains a strong discursive dimension [14]. A study on the discursive representations of older people will provide insight into the kinds of socially shared patterns of thought and behavior [14] that govern attitudes toward members of this group.

Our study is significant in several ways. Conceptually, this is one of the first known studies to use large-scale and multi-sourced databases to provide a comprehensive view of ageism in the US and UK. Previous content analyses of societal ageism are mostly singled-sourced (e.g., poetry or fiction)—more details elaborated on below. We chose the US and UK as they are home to the world’s largest media conglomerates [15] and therefore have tremendous power to shape public opinion [11]. Practically, the detailed and timely content analysis of ageism enables targeted messaging to counteract it. Of broader significance, this study creates an unprecedented platform for social scientists and policy makers to study societal perceptions that complements traditional methods of focus groups and surveys. We synthesize the literature on the impact of age stereotypes on well-being and the conceptual framework of age stereotypes.

### 1.1. Impact of Aging Stereotypes in Physical and Mental Well-Being

Levy and Langer [16] investigated attitudes towards aging and the effects of negative aging stereotypes on memory loss in American deaf and older adults in China who were previously unexposed or minimally exposed to negative age stereotypes from the media, and hearing Americans who are constantly exposed to negative aging stereotypes through various media channels [17]. As predicted, the Chinese participants reported the most positive attitudes toward aging, following by the American deaf and the American hearing sample. The same rank order was also observed in the memory scores of these three groups. These results show that negative aging stereotypes have a detrimental effect on memory.

A survey of older adults from 65–95 years found that negative age stereotypes were associated with decreasing self-reported sense of responsibility toward others, subjective health, adherence to medical appointments, participation in community social activities, and regular physical activity [18]. Overall, negative aging stereotypes were associated with lower overall quality of life for older adults.

On the other hand, positive aging stereotypes resulted in a variety of positive outcomes. Researchers primed either negative or positive stereotypes in a group of older adults above 62 years and put them through mathematical and verbal tasks [19]. The positive stereotype group recorded significantly lower cardiovascular stress as evidenced by lower systolic and diastolic blood pressure, heart rate, and skin conductance. Another study found that older adults with more positive age stereotypes had better screened hearing after 36 months [20]. Positive age stereotypes were also linked to 7.5 years of life among participants of the Ohio Longitudinal Study of Aging and Retirement compared to those who espoused negative views [21]. In essence, these landmark studies found that negative age stereotypes are linked to poorer physical and psychological health, highlighting the importance of studying the media origins of these stereotypes to counteract them.

### 1.2. Conceptual Framework and Measurement of Age Stereotypes

We synthesize the extant literature on the conceptual framework and measurement of age stereotypes to highlight the need for more rigorous datasets and methods that our study provides. We build on the conceptual framework that the multiple dimensions of age stereotypes can be grouped by valence and theme [22,23]. With regard to valence, age stereotypes can either be positive or negative. For example, travel advertisements typically portray negative images of grumpy and listless older adults who could be transformed into beaming “golden agers” (positive image) basking in the Florida sun by buying their packages [24]. With respect to theme, age stereotypes often involve physical and/or behavioral attributes [25]. For example, companies often portray older individuals as suffering from physical problems, such as the inability to perform activities of daily living, to market their supplements and solutions [26].

On the measurement front, direct and indirect strategies are used. Direct methods include interviews and surveys methods where participants are asked directly about their perceptions through telephone interviews [27], established scales like the Palmore Fact of Aging Quiz [28], and Implicit Association Tests (IAT) to understand stereotypical associations at the level of automatic cognition [29]. A key study across 26 countries/territories surveyed 3435 college students and found that negative perceptions of older adults are similar across different countries in the domains of physical attractiveness, ability to perform everyday tasks, and learning new things [30].

While Direct Method studies have contributed significantly to understanding age stereotypes, there are limitations. Firstly, survey studies sometimes use convenience sampling of college students to draw country-level conclusions—the ecological fallacy [31,32]. Secondly, survey studies typically use validated scales that were designed years ago, capturing the content of ageism at the time they were designed, and may not reflect the current reality. The broader point is the need for indirect methods that take a bottom-up approach of capturing the content of age stereotypes, to complement the top-down approach of validated scales.

Indirect methods to investigate ageism include ethnographic approaches [33], analysis of fiction [34], images from literature [35], poetry [36], and art [37]. For example, a study examined 150 age-specific birthday cards and found that textual messages tend to be more ageist than pictures [38]. Of the cards with textual messages, 66.7 percent represented ageing negatively. There are allusions to cognitive decline like “Oh you’re 50? You don’t even remember what I’m talking about do you? Oh well, happy 50th birthday anyway”. Other cards, though whimsical, sarcastically scorned physical decline “As you’re entering your 50s new doors will open for you. Geriatric crisis center, cosmetic surgery clinic, office of ageing.” In-depth interviews also found that ageism existed in the advertising industry, with older workers perceived by young workers as “dead wood” [39]. Overall, the results are remarkably similar: Negative stereotypes are highly prevalent, compared to positive stereotypes. Against this backdrop, we hypothesize that negative age stereotypes will outnumber positive/neutral ones (Hypothesis 1), as shown in previous studies, albeit single sourced.

There are nuances across sources. Older men are hardly present in top men’s magazines in the US; women’s magazines feature mainly younger women, although older women form the majority of their readers [40,41]. In the UK, advertisements in magazines that target an older audience typically associate older adults with food items and hearing aids. However, positive stereotypes surface occasionally in magazines; for instance, older adults were portrayed as sexually attractive with ‘optimal sexual engagement’ in the dating sections of Canadian magazines [42], though magazines typically portrayed older adults negatively in both content [43] and advertisements [44]. We hypothesize that among popular genres (spoken/TV, fiction, popular magazines, newspapers), magazines contain the highest ageism scores (Hypothesis 2).

These indirect and bottom-up methods are helpful in understanding societal ageism as cultivation theory states that different forms of media reflect societal perceptions they seek to portray [45]. However, most studies are single-sourced, anecdotal, and run the risk of cherry-picking sources to support a scientific agenda [46]. Importantly, single-sourced studies do not provide strong evidence for a targeted media campaign at the societal level. The focus on single sources is likely due to the lack of suitable large-scale datasets and methods to interrogate them. Recent grants by the National Endowment for the Humanities (NEH) and the National Science Foundation (NSF) provided unprecedented corpora, and scholars have adapted computational linguistics methods to analyze these databases for societal stereotypes [47,48,49,50,51,52], which we will leverage for the current study.

## 2. Materials and Methods

### 2.1. Datasets

The British National Corpus (BNC) is compiled by Oxford University’s Computing Services. It is a 100-million-word corpus that spans the 1980s to 1993 and represents a wide cross-section of British English, both spoken and written. The written part of the BNC (90%) includes extracts from regional and national newspapers, specialist periodicals and journals for all ages and interests, academic books, popular fiction, published and unpublished letters and memoranda, school and university essays, among many other kinds of text. The spoken part (10%) consists of orthographic transcriptions of unscripted informal conversations (recorded by volunteers selected from different age, region, and social classes in a demographically balanced way) and spoken language collected in different contexts, ranging from formal business or government meetings to radio shows and phone-ins. Work on building the corpus began in 1991, culminating in the latest third edition BNC XML in 2007.

The Corpus of Contemporary American English (COCA) is the largest, balanced corpus of contemporary American English. It contains more than one billion words of text, including 20 million words each year from 1990, and it is equally divided among spoken, fiction, popular magazines, newspapers, and academic texts. The even distribution of various sources gives the corpus its balance. The corpus is also updated every six to nine months, therefore serving as a unique record of linguistic changes in American English [53].

### 2.2. Measurement of Age Stereotypes

We analyzed the prevalence of various synonyms in the datasets. ‘Elderly’ is the most commonly used synonym with the highest prevalence of 22.2 per million. Other synonyms evidenced a markedly lower prevalence, for example, ‘old people’ had a prevalence of 0.72 per million. This imbalance corroborated previous findings that older adults are labelled negatively in the media [51]. While we acknowledge the negative connotation of ‘elderly’, and the movement against it [54], it was used as a target synonym as it provided sufficient data for meaningful analysis. This however presents limitations that we address in the paper.

We compiled the top 20 words that co-occurred most frequently with the target word, known as collocates, with the following inclusion criteria: (a) Lexical Proximity: collocate present within four words prior or after the target word. Articles such as ‘the’, ‘a’ were not included in the six-word lexical span. If the target noun was the first word of a sentence, the collocates from the prior sentence were excluded.; (b) Relevant context: collocate referred to specifically to an old person (checked by two raters); (c) Mutual Information (MI) Score of three and above: collocate had a stronger association with the respective synonym than other words in the corpus indicating semantic bonding [50,55]. MI score estimates word association norms directly from the corpus. It is calculated via sentiment analysis, which shows the mutual information between collocates and target words. The higher the MI value, the closer the relationship between the collocate and target word. The MI value is calculated using the formula:

$$\mathrm{MI}=\frac{\log\left[\frac{C\times SizeCorpus}{A\times B\times Span}\right]}{\log2}$$

‘*A*’ indicates the possibility of the target word A appearing, which is calculated by the frequency of the target word. ‘*B*’ indicates the possibility of the collocate B appearing, which is calculated by the frequency of word B. ‘*C*’ indicates the possibility of ‘*A*’ and ‘*B*’ appearing together, which is calculated by the frequency of collocate B appearing near the target word A. ‘*SizeCorpus*’ refers to the size of corpus or the number of words. Span is the span of words (e.g., if there are 6 words to the left and 6 words to the right of the target word, span = 12). log (2) = 0.30103. This is a well-established application of computational linguistics to study stereotypes in other studies [47,48,49,50,51,52].

Thereafter, each collocate that met the study criteria was rated in binary fashion: 1 (negative) and 0 (positive/neutral) to test Hypothesis 1. Each collocates was also rated on a scale from 1 (very positive) to 5 (very negative) for Hypothesis 2—a method found to be valid and reliable to measure age-stereotype associated words [16]. For example, ‘frail’ and ‘dementing’ are rated as very negative while ‘caring’ and ‘happy’ are rated as very positive. Ratings were done by two independent gerontology researchers—consistent with similar corpus studies [47,48,49,50,51,52]—and the inter-rater reliability using Cronbach’s alpha was 0.972 (95% CI: 0.946, 0.986). Age stereotype scores were created by calculating the mean of all scores for the respective corpora using this mixed-method approach that integrated the rigor of text analytics and qualitative depth as exemplified in the coding process. Table 1 presents sample descriptors of older adults that are negative, neutral, or positive.

### 2.3. Analytic Strategy

To test Hypothesis 1—negative age stereotypes will outnumber positive/neutral ones—we ran a chi-square goodness-of-fit to show that the actual distribution of negative age stereotypes was significantly different from the expected distribution. Hypothesis 2 stated that among popular genres (spoken/TV, fiction, magazines, newspapers), magazines contain the most negative age stereotype scores. We ran a two-way ANOVA to test the interaction between corpus and genre; specifically, the main effect of genre will be used to test Hypothesis 2. All data pre-processing, text analytics and statistical analyses were conducted in Python 3.7. and OriginPro 2019b.

## 3. Results

The size of conversations in the UK about older adults (48.96 words per million) is significantly higher compared to the US (27.16 words per million), *T*_7_ = −3.77, *p* = 0.007. In the UK, the highest prevalence of conversations related to older adults appeared in newspapers at 60.96 words per million while in the US, it was magazines at 30.99 words per million. As hypothesized, a chi-square goodness-of-fit achieved significance, *X*^2^ (1) = 5.44, *p* = 0.02, showing that the actual distribution of negative age stereotypes was significantly different from the expected distribution. Across the genres, there are six times more negative descriptors of older adults compared to positive/neutral ones, supporting Hypothesis 1. Table 2 shows the top 10 descriptors/collocates of older adults.

The country and genre interaction effect did not reach significance. US evidenced significantly higher negative stereotypes than UK in the corpus, *F*(1, 28) = 4.75, *p* = 0.038. As hypothesized, ageism across genre also reached significance, *F*(3, 28) = 4.69, *p* = 0.009, with the highest ageism in magazines (M = 4.80, SD = 0.45), followed by spoken (M = 4.60, SD = 0.74) and newspapers (M = 4.35, SD = 1.33), with the least in fiction (M = 3.00, SD = 1.27), providing support for Hypothesis 2.

## 4. Discussion

This study used large-scale (1.1 billion words) and multi-sourced genres of spoken/TV, fiction, magazines, newspapers to provide a comprehensive view of ageism in the US and UK and employed a technique that represents new advances in the study of aging narratives and stereotypes. Collectively, both countries are home to the world’s largest media conglomerates [15] and have outsized power to shape public opinion [11]. Specifically, we contributed to the indirect method of analyzing age stereotypes that have been primarily single-sourced. The key finding is consistent, though no less alarming: Negative age stereotypes outnumber positive ones by six times. Negative stereotypes tend to be physical (e.g., frail), while positive ones tend to be behavioral (e.g., caring). Magazines contained the highest levels of ageism, followed by the spoken genre (e.g., TV and radio talk shows).

The content of negative age stereotypes in the present study, replicated findings from many single-sourced studies such as fiction [34], art [37], and poetry [36]. The prevalence of negative age stereotypes could be a result of coverage bias where negative events and narratives tend to gain more attention than positive ones [56]. In any case, the prevalence of negative age stereotypes across all media genres of our dataset is instructive of society’s perceptions of ageing as the media reflect societal perceptions they seek to portray [40,45].

Our study made the following contributions. First, negative social realities of ageing are widespread—across multiple media genres—and not only confined within advertisements or fiction. Ageism in the traditional media like newspapers could have an amplification effect as the negative stereotypes are funneled through the echo chambers of social media platforms that draw widely from these news sources. Drilling further, the prevalence of ageism across magazines and newspapers signals the possibility of an implicit age bias in journalists. While scholars have looked at how implicit racial [57] and gender bias [58] can emerge in the reporting and writing process, little is known about whether journalists carry implicit age biases—an area that warrants further scrutiny in research. Nonetheless, evidence suggests that journalists who are aware of their cognitive biases tend to produce less biased work than those who are not [58]. Self-awareness exercises could be held to ensure that journalists make the conscious effort to curb their age biases to prevent inaccurate stereotypes from being perpetuated. This is particularly crucial given that newspapers and magazines hold a discursive power to propagate ageism.

The fact that magazines contain the highest levels of ageism has serious consequences for the older demographic. A recent study revealed a strong discrepancy in print magazine readership by age, with individuals aged 65 and above twice as likely to read a print magazine as those aged from 18 to 24 in the United States [59]. The constant exposure to damaging views of aging is likely to result in self-limiting views of the aging process, which will affect the health of older adults [12].

Thus far, interventions to reduce ageism have been designed at both the interpersonal and societal level. Significant progress has been made at the interpersonal level [59]. However, at the societal level, campaigns to combat the overwhelmingly negative portrayals of older adults in the media are few, with the most recent being ‘Disrupt Aging Collection’. This campaign presented realistic and positive images of aging and was launched by the American Association of Retired Persons (AARP) in collaboration with Getty Images [60].

The effectiveness of such laudable media campaigns ultimately depends on the ability to target specific erroneous and negative stereotypes. However, the current state of research regarding media portrayals of older adults is typically single-sourced and does not provide strong evidence for targeted campaigns. Furthermore, one of the most common and insidious ways that ageism is expressed is through language [61]. As exemplified by the viral phrase ‘Ok, Boomer’, age stereotypes constantly take on new forms—be it through new slurs or lingo. Prejudice cannot be alleviated or understood fully without paying attention to how stereotypes are embedded in language [62]. As such, it is imperative that policy makers are cognizant of how age stereotypes evolve and manifest in new ways.

Practically, interventions and awareness campaigns could focus on educating journalists as their prose wields significant power to influence societal attitudes. Of broader significance, the study provides a roadmap on where and how to focus anti-ageism campaigns. Prior research has established that educational programs have the potential to reduce ageism [63]. Both mass media and societal campaigns could therefore include an educational component to equip members of the public with a more accurate understanding of older adults and aging in general. Societal campaigns could target magazines and TV where ageism levels are the highest by framing aging in a way that takes into account both the gains and losses inherent in the aging process [52]. Although a certain number of older adults are at risk of becoming frail during their later years, there is also evidence indicating that overall physical functioning of older adults has improved over time [64]. Likewise, even as old age poses a severe risk for COVID-19 mortality, there have been occasions where older adults have recovered from the virus and younger people have not [4]. Thus, both positive and negative narratives of older people should be featured in mainstream discourse to ensure a more balanced portrayal of the aging process.

From a methodological standpoint, our study provided a proof-of-concept for scholars and policy makers to study societal narratives using corpus data, which complements traditional methods of focus groups and surveys [65,66,67,68,69,70,71,72,73]. There is potential for future studies using our datasets. For example, in this COVID-19 pandemic, techniques described in this study could be used to track the societal narratives [74] around vulnerable groups for ageism and racism. Understanding the content of ageism and racism during a pandemic provides important evidence for designing a targeted campaign to counteract it.

While we sought to circumvent the weaknesses of other studies by providing a multi-sourced platform of 1.1 billion words, this study is not without weaknesses. We did not include social media in our corpus as most major platforms (e.g., Facebook) no longer have open and publicly available data, leaving limited and outdated datasets that may not be comprehensive. Nevertheless, this is a significant weakness as ageism cannot be compared across media genres. A future iteration of the study could include social media for comparative analysis to facilitate advocacy campaigns.

Another issue is the use of ‘elderly’ as a target search term. The term has negative connotations and would therefore attract negative collocates. While we are cognizant of the negative connotations and wholeheartedly support the movement against it [54], our decision was based on data considerations. ‘Elderly’ was the most common synonym with the highest prevalence of 22.2 per million, over 30 times more than ‘old people’ with a low prevalence of 0.72 per million. Choosing the latter would not generate a sufficient dataset for analysis. Nevertheless, it is unfortunate that a derogatory synonym is the most commonly used term for an older adult and future studies should focus on strategies to reframe ageing positively in the media.

## 5. Conclusions

In conclusion, this study contributed by detailing how ageism manifests across different genres of media. We found support for our hypotheses that negative age stereotypes in the media outnumber those that are positive or neutral, and that magazines contain the most ageist content. There is an urgent need to study the content of ageism such that societal campaigns can be designed to combat it. We hope to have laid the groundwork to design interventions to tackle societal ageism—one of our generation’s most insidious threats.

## Figures and Tables

**Table 1:** Examples of Negative, Neutral and Positive Descriptors of Older Adults in the Media.

Negative	Neutral	Positive
abuse	society	acclaimed
ailing	bus	appreciate
bad	transport	cheerful
frail	group	wisdom
broken	kitchen	blessing
burden	church	valuable
weak	train	charitable
Dead	clothing	happy
tragedy	golf	venerable
senile	seat	encouraging

**Table 2:** Top 10 Descriptors of Elders in the UK and US in a 1.1-billion-word corpus of popular media.

Top Descriptors of Elders in the UK ^1^	Top Descriptors of Elders in the US ^2^
Frail	Hoarders
Infirm	Community-dwelling
Housebound	Infirm
Dementing	Homebound
Long-stay	Frail
Disabled	Disabled
Spinster	Functional
Ageing	Indigent
Childless	Impaired
Vulnerable	Handicapped

Notes*:* ^1^ Top collocates of “elderly” in the British National Corpus ranked by mutual information score. ^2^ Top collocates of “elderly” in the Corpus of Contemporary American Corpus ranked by mutual information score.

## Data Availability

Data are publicly available online at https://www.english-corpora.org (accessed on 22 July 2021).
